# P-1773. Intestinal Parasitic Infections in Japan: Cross-sectional Study of Prevalence and Risk Factors in Suspected Infectious Gastroenteritis

**DOI:** 10.1093/ofid/ofaf695.1943

**Published:** 2026-01-11

**Authors:** Akira Kawashima, Megumi Akashi, Yusuke Oshiro, Yasuaki Yanagawa, Rieko Shimogawara, Masami Kurokawa, Naokatsu Ando, Haruka Uemura, Takahiro Aoki, Kei Yamamoto, Junichi Akiyama, Daisuke Mizushima, Kenji Yagita, Hiroyuki Gatanaga, Koji Watanabe

**Affiliations:** National Center for Global Health and Medicine, Japan Institute for Health Security, Shinjuku-ku, Tokyo, Japan; National Center for Global Health and Medicine, Japan Institute for Health Security, Shinjuku-ku, Tokyo, Japan; National Center for Global Health and Medicine, Japan Institute for Health Security, Shinjuku-ku, Tokyo, Japan; National Center for Global Health and Medicine, Japan Institute for Health Security, Shinjuku-ku, Tokyo, Japan; National Institute of Infectious Diseases, Japan Institute for Health Security, Shinjuku-ku, Tokyo, Japan; National Center for Global Health and Medicine, Japan Institute for Health Security, Shinjuku-ku, Tokyo, Japan; National Center for Global Health and Medicine, Japan Institute for Health Security, Shinjuku-ku, Tokyo, Japan; National Center for Global Health and Medicine, Japan Institute for Health Security, Shinjuku-ku, Tokyo, Japan; National Center for Global Health and Medicine, Japan Institute for Health Security, Shinjuku-ku, Tokyo, Japan; National Center for Global Health and Medicine, Japan Institute for Health Security, Shinjuku-ku, Tokyo, Japan; National Center for Global Health and Medicine, Japan Institute for Health Security, Shinjuku-ku, Tokyo, Japan; National Center for Global Health and Medicine, Japan Institute for Health Security, Shinjuku-ku, Tokyo, Japan; National Institute of Infectious Diseases, Japan Institute for Health Security, Shinjuku-ku, Tokyo, Japan; National Center for Global Health and Medicine, Japan Institute for Health Security, Shinjuku-ku, Tokyo, Japan; Tokai University School of Medicine, Isehara-shi, Tokyo, Japan

## Abstract

**Background:**

In developed countries, intestinal parasites are generally considered as an agent occurring among people who are traveling to or immigrants from endemic area with poor sanitation. Also, recent epidemiological studies showed that *Entamoeba histolytica* could happen as sexually transmitted infection (STI). However, the epidemiology of the other intestinal parasite infestations (IPIs) is still unclear in many developed countries.

**Figure 1.**
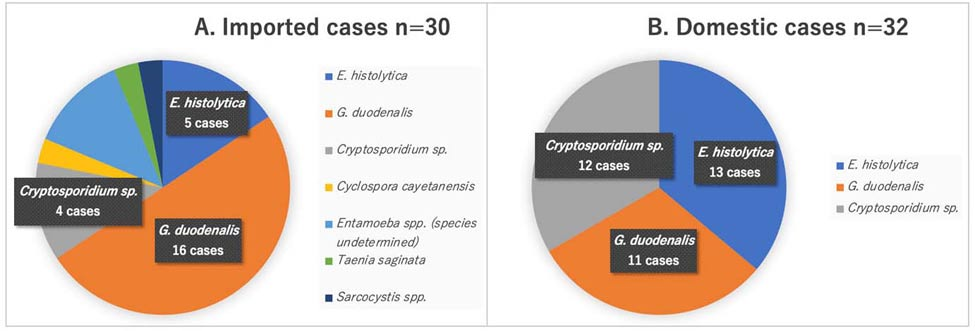
Distribution of intestinal parasites. Intestinal parasitic infections were determined by modified O&P in the present study. (A) Intestinal parasites seen in imported cases were presented, in which multiple parasites were detected from one sample in two cases. (B) Intestinal parasites seen in domestic cases are presented, in which multiple parasites are detected from one sample in four cases.

**Table 1.**
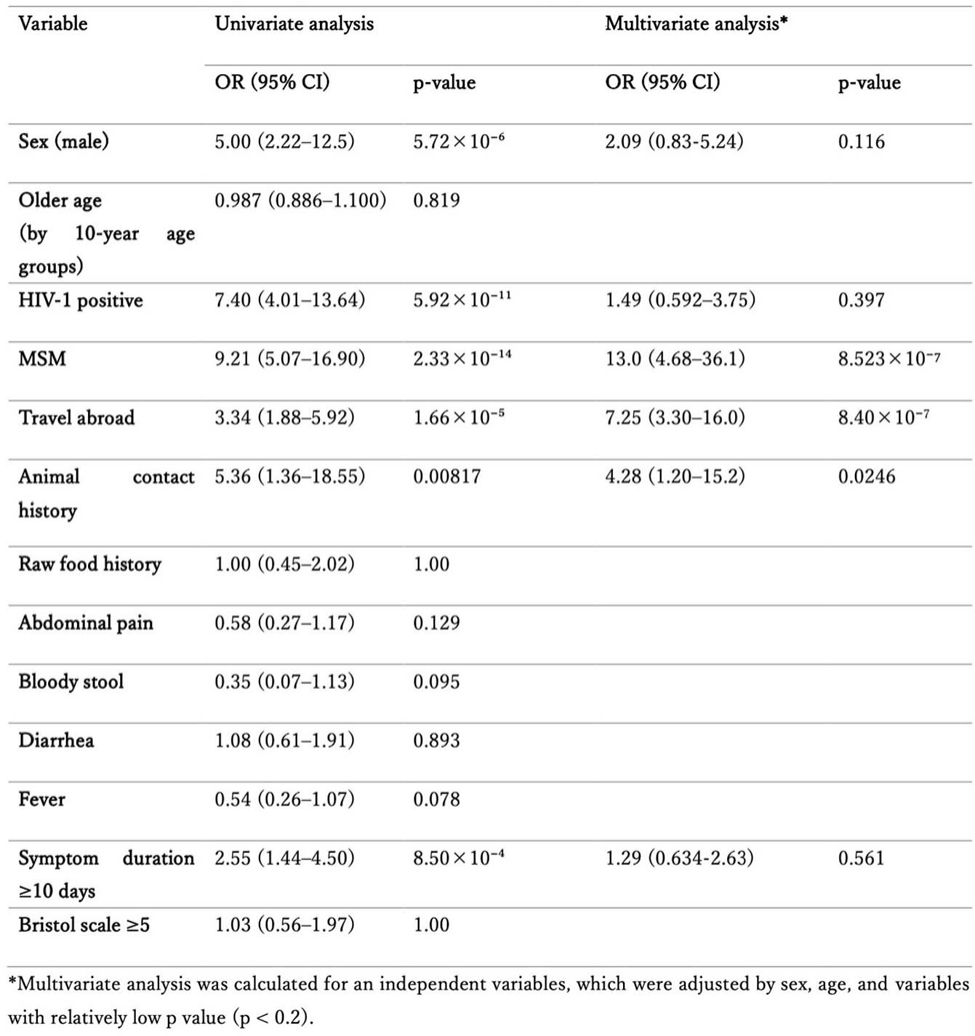
Impact of patients’ characteristics and symptoms on the incidence of IPIs. *Multivariate analysis was calculated for an independent variables, which were adjusted by sex, age, and variables with relatively low p value (p < 0.2).

**Methods:**

For stool samples from the patients with suspicion of infectious gastroenteritis, modified stool ova and parasite examination (modified O&P) was performed as following. Firstly, stool samples are mixed with fluorescent conjugated antibodies against *Giardia* cyst, and *Cryptosporidium* oocyst, thereafter, the sample is examined by both fluorescent microscopy and bright-field microscopy. It has compatibly high sensitivity as antigen detection test or PCR for the detection of these protozoa.

**Results:**

During 3-year study period, 624 stool samples were examined by modified O&P. IPIs were confirmed in 62 cases (9.9%); 30 as imported cases and 32 as domestic cases (Fig. 1). The most common parasites were *G. duodenalis*, *E. histolytica*, and *Cryptosporidium* spp. Interestingly, domestic parasite infections were limited to these three protozoa, in which four cases showed multiple protozoa.

In contrast, various types of parasites were detected in imported cases. Multivariate logistic regression identified not only recent travel history to endemic area but also men who have sex with men and animal contact as independent risk factors for IPIs (Table 1).

**Conclusion:**

*G. duodenalis* and *Cryptosporidium spp*. are commonly reported as domestic cases, whose frequency was the same level as that of *E. histolytica*. Also, the results from regression analysis suggest that these protozoa are spreading as sexually transmitted infection among MSM, and zoonoses. Molecular epidemiological study is currently underway. Active epidemiological surveillance is warranted in the other developed countries.

**Disclosures:**

All Authors: No reported disclosures

